# Kunitz-Type Peptides from the Sea Anemone *Heteractis crispa* Demonstrate Potassium Channel Blocking and Anti-Inflammatory Activities

**DOI:** 10.3390/biomedicines8110473

**Published:** 2020-11-04

**Authors:** Irina Gladkikh, Steve Peigneur, Oksana Sintsova, Ernesto Lopes Pinheiro-Junior, Anna Klimovich, Alexander Menshov, Anatoly Kalinovsky, Marina Isaeva, Margarita Monastyrnaya, Emma Kozlovskaya, Jan Tytgat, Elena Leychenko

**Affiliations:** 1 G.B. Elyakov Pacific Institute of Bioorganic Chemistry, Far Eastern Branch, Russian Academy of Sciences, 159, Pr. 100 let Vladivostoku, 690022 Vladivostok, Russian; sintsova0@gmail.com (O.S.); annaklim_1991@mail.ru (A.K.); almenshov1990@gmail.com (A.M.); kaaniw@piboc.dvo.ru (A.K.); issaeva@gmail.com (M.I.); rita1950@mail.ru (M.M.); kozempa@mail.ru (E.K.); 2 Toxicology and Pharmacology, University of Leuven (KU Leuven), Campus Gasthuisberg O&N2, Herestraat 49, P.O. Box 922, B-3000 Leuven, Belgium; steve.peigneur@kuleuven.be (S.P.); ernestolopesjr@gmail.com (E.L.P.-J.); jan.tytgat@kuleuven.be (J.T.)

**Keywords:** sea anemone, Kunitz fold, type 2 potassium channel toxins, electrophysiology, anti-inflammatory activity

## Abstract

The Kunitz/BPTI peptide family includes unique representatives demonstrating various biological activities. Electrophysiological screening of peptides HCRG1 and HCRG2 from the sea anemone *Heteractis crispa* on six Kv1.x channel isoforms and insect *Shaker* IR channel expressed in *Xenopus laevis* oocytes revealed their potassium channels blocking activity. HCRG1 and HCRG2 appear to be the first Kunitz-type peptides from sea anemones blocking Kv1.3 with IC_50_ of 40.7 and 29.7 nM, respectively. In addition, peptides mainly vary in binding affinity to the Kv1.2 channels. It was established that the single substitution, Ser5Leu, in the TRPV1 channel antagonist, HCRG21, induces weak blocking activity of Kv1.1, Kv1.2, and Kv1.3. Apparently, for the affinity and selectivity of Kunitz-fold toxins to Kv1.x isoforms, the number and distribution along their molecules of charged, hydrophobic, and polar uncharged residues, as well as the nature of the channel residue at position 379 (Tyr, Val or His) are important. Testing the compounds in a model of acute local inflammation induced by the introduction of carrageenan administration into mice paws revealed that HCRG1 at doses of 0.1–1 mg/kg reduced the volume of developing edema during 24 h, similar to the effect of the nonsteroidal anti-inflammatory drug, indomethacin, at a dose of 5 mg/kg. ELISA analysis of the animals blood showed that the peptide reduced the synthesis of TNF-α, a pro-inflammatory mediator playing a leading role in the development of edema in this model.

## 1. Introduction

Peptides of the Kunitz/BPTI family contain one of the most evolutionarily ancient and conserved structural motifs, i.e., the Kunitz fold, which is widely distributed among both venomous terrestrial and marine organisms [1]. Historically, the firstly discovered representative of this family, the bovine pancreatic trypsin inhibitor (BPTI) [2], is known as an inhibitor of different serine proteases and capable of carrying out an anti-inflammatory function participating in proliferation and angiogenesis [3,4,5]. Kunitz-type peptides from snake, spider, scorpion, and sea anemone venoms are encoded by multigene families and form combinatorial libraries of homologous peptides [6,7,8,9,10]. Differing by single amino acid substitutions, some of these peptides not only exhibit protease inhibitory activity, but also can block voltage-gated potassium (Kv) [10,11,12,13,14,15,16,17,18,19], calcium (Cav) [20], acid-sensing ion (ASIC) channels [21], and transient receptor potential vanilloid 1 (TRPV1) [22,23,24]. Furthermore, some of them can interact with integrins [25] and vasopressin receptor 2 [26] as well.

Sea anemone Kv toxins are represented by six unique peptide folds: ShK (type 1), Kunitz-domain (type 2), β-defensin-like (type 3), boundless β-hairpin (type 4), an unknown fold predicted to form an inhibitor cystine knot (type 5), and the PHAB fold (type 6) [27,28]. Type 2 toxins, having the Kunitz fold, include κ1.3-ATTX-As2a-c (AsKC1–AsKC3 or kalicludines 1–3) from *Anemonia sulcata* [16], APEKTx1 from *Anthopleura elegantissima* [17], κ1.3-SHTX-Sha2a (SHTX III) from *Stichodactyla haddoni* [18], and ShPI-1 from *Stichodactyla helianthus* [19]. Kalicludines block Kv1.2 in µM concentrations and strongly inhibit trypsin [16]. APEKTx1, similarly to dendrotoxins (DTX), selectively blocks Kv1.1 channels along with effectively inhibiting trypsin [17]. It was determined that SHTXIII also inhibited trypsin and competed with the binding of α-DTX (alpha-dendrotoxin from mamba snake) in rat synaptosomal membranes at the level of Kv1.1, Kv1.2, and Kv1.6 channels [18]. Since SHTXIII paralyzes crabs, it can block not only mammalian but also crustacean Kv channels [18]. A pseudo wild-type variant of the natural peptide ShPI-1, rShPI-1A, known as an inhibitor of serine, aspartate, and cysteine proteases, was shown to bind to Kv1.1, Kv1.2, and Kv1.6 channels with IC_50_ values in the nM range [19]. Therefore, all known type 2 toxins block Kv channel subtypes widely expressed in neurons of the central nervous system (CNS), in particular Kv1.1, Kv1.2, and Kv1.6 [29].

In contrast to type 2, toxins of types 1, 3, and 5 [30,31] block also the Kv1.3 channelsubtype. Since Kv1.3 channels are expressed in the CNS and immune cells, including microglia, dendritic cells, T (TEM cells), B lymphocytes, and macrophages [32], they mediate autoimmune diseases [33], participate in chronic inflammatory diseases and cancer progression (due to its double role in proliferation and apoptosis regulation) [34]. The most selective toxin ShK from *S. helianthus* with high affinity to Kv1.3 channel (11 pM) [35] has been the subject of intense research, both fundamental and clinical [30,31]. Its designed analog, ShK-186 (Dalazatide), is now undergoing clinical trials on psoriasis [36]. Blocking of Kv1.3 channels decreases the expression levels of pro-inflammatory mediators and may be used in many conditions like neurodegenerative, autoimmune, and chronic diseases accompanied by inflammation. It has been established that the treatment of autoreactive T-lymphocytes by ShK-186 decreases the levels of IL-2, interferon-γ, TNF-α, and IL-4 [37]. Therefore, peptide inhibitors of Kv1.x channels can be assumed as promising compounds for drug design, as well as valuable tools for the investigation of these channels.

Here we report an in vivo anti-inflammatory activity and potassium channel blocking the activity of Kunitz peptides of the sea anemone *Heteractis crispa* by the electrophysiological screening on six isoforms of Kv1.x channels and insect *Shaker* IR channel expressed in *Xenopus laevis* oocytes.

## 2. Experimental Section

### 2.1. Purification and Characterization

The native peptides HCRG1 and HCRG2 were isolated as described in [38]. In brief, the peptides were precipitated from a water extract of the sea anemone *Heteractis crispa* (collected by dredging) with 80% acetone; next gel filtration chromatography on an Akrilex P-4 column was carried out, followed by cation-exchange chromatography on a CM-32 cellulose column, with a final purification step using an RP-HPLC Nucleosil C18 column.

The mutant peptide HCRG21 S5L was made using the QuikChange^®^ Site-Directed Mutagenesis Kit (Stratagene, La Jolla, CA, USA), based on the wild type plasmid pET32b-HCRG21 using gene-specific primers (dir 5′-CGTGGTATCTGCTTAGAACCGAAAGTTG-3′; rev 5′-CAACTTTCGGTTCTAAGCAGATACCACG-3′). The resulting mutant plasmid was verified by DNA sequencing, and the target peptide was expressed and purified using the same conditions as was reported for the recombinant peptide HCRG21 [23]. The target peptide was isolated by HPLC on a Jupiter C4 column (10 × 250 mm, Phenomenex, Torrance, CA, USA), equilibrated by 0.1% TFA, pH 2.2, and eluted in gradient of acetonitrile concentration (Solution B) for 70 min at 3 mL/min.

### 2.2. MALDI-TOF/MS Analysis

MALDI-TOF/MS spectra of peptides were recorded using an Ultra Flex III MALDI-TOF/TOF mass spectrometer (Bruker, Bremen, Germany) with a nitrogen laser (SmartBeam, 355 nm), reflector and potential LIFT tandem modes of operation. Sinapinic acid was used as a matrix. External calibration was employed using a peptide InhVJ with m/z 6107 [39] and its doubly-charged variant at m/z 3053.

### 2.3. NMR Spectroscopy

The NMR spectrum was acquired at 30 °C on a Bruker Avance III 700 MHz spectrometer (Bruker Biospin, Billerica, MA, USA) equipped with a triple resonance z-gradient TXO probe. Peptide HCRG21 S5L was dissolved in 90% H_2_O/10% D_2_O (Deutero GmbH, Kastellaun, Germany) at a concentration of 2 mg/mL. Excitation sculpting with gradients [40] was applied to suppress strong solvent resonance, the chemical shift of their signal was arbitrary chosen as 4.7 ppm. TopSpin 3.6 (Bruker Biospin, Billerica, MA, USA) was used for spectrum acquisition and processing.

### 2.4. Inhibitory Activity

The trypsin inhibitory activity of HCRG21 S5L was tested through the standard procedure [41] using N-α-benzoyl-D,L-arginine p-nitroanilide hydrochloride (BAPNA). Determination of the peptide trypsin inhibition constant was performed according to the method of Dixon [42] using substrates concentrations of 0.1, 0.16, and 0.256 mM. The concentration of trypsin in the reaction mixture was 50 nM and the tested peptide ranged from 0 up to 512 nM. The inhibitory constants were calculated based on the results of three parallel experiments.

### 2.5. Expression of Voltage-Gated Ion Channels in Xenopus laevis Oocytes

For the expression of rKv1.1, hKv1.2, hKv1.3, rKv1.4, rKv1.5, rKv1.6, and *Shaker* IR in *Xenopus laevis* oocytes, the linearized plasmids were transcribed using the T7 or SP6 mMESSAGE-mMACHINE transcription kit (Ambion, Austin, TX, USA). The harvesting of stage V–VI oocytes from anaesthetized female *X. laevis* frog was as previously described [43]. Oocytes were injected with 50 nL of cRNA at a concentration of 1 ng/nL using a micro-injector (Drummond Scientific, Broomall, PA, USA). The oocytes were incubated in a solution containing (in mM): NaCl, 96; KCl, 2; CaCl_2_, 1.8; MgCl_2_, 2; and HEPES, 5 (pH 7.4), supplemented with 50 mg/L gentamicin sulfate.

### 2.6. Electrophysiological Studies

The physiological activity in oocytes expressing heterologously the voltage-gated ion channel proteins was determined by the two-electrode voltage-clamp technique, using a Geneclamp 500 amplifier (Molecular Devices, Austin, TX, USA) controlled by the pClamp database system (Axon Instruments, Union City, CA, USA). The measurements were performed at room temperature (18–22 °C). Whole-cell currents were recorded 1–4 days after the mRNA injection. The electrode resistance was 0.7–1.5 MΩ. The signal was amplified, preliminarily filtered by the amplifier embedded four-polar Besselian filter (cutoff frequency 500 Hz) after digitization of the signal at 2000 Hz. Recordings obtained before the activation of the examined currents were used for subtraction of the capacitive and leakage current. The cells were kept at a holding potential of −90 mV. The membrane potential was depolarized to 0 mV for 250 ms with a subsequent pulse to −50 mV for 250 ms in the case of the Kv1.1–Kv1.6 and *Shaker* channels. For statistical analysis, the Student’s coefficient (*P* < 0.05) was used. All the results were obtained from at least three independent experiments (n ≥ 3) and are expressed as mean value ± standard error. The use of the *X. laevis* animals was in accordance with the license number LA1210239 of the Laboratory of Toxicology and Pharmacology, University of Leuven (Belgium). The use of *X. laevis* was approved by the Ethical Committee for animal experiments of the University of Leuven (P186/2019). All animal care and experimental procedures agreed with the guidelines of the European Convention for the protection of vertebrate animals used for experimental and other scientific purposes (Strasbourg, 18.III.1986).

### 2.7. Acute Toxicity of HCRG1

The animal studies were performed under the European Convention for the human methods for the animal welfare (Directive 2010/63/EU), the National Standard of the Russian Federation “Good Laboratory Practice” (GOST P 53434-2009, Russia), and was approved by G.B. Elyakov Pacific Institute of Bioorganic Chemistry (Far Eastern Branch, Russian Academy of Sciences) Committee on Ethics of laboratory animal handling 2017/78-A protocol. Adult male ICR line white mice weighing 20–22 g were kept at room temperature with a 12-h light/dark cycle and with ad libitum access to food and water.

HCRG1 was administered once intravenously at doses of 0.1 and 1 mg/kg, control group received saline (0.9% NaCl) (10 mL/kg or 0.250 mL/mouse). Six mice in each group were used. Then, changes in basic physiological parameters, such as motility, behavioral responses, and physical activity, were registered in each group of animals within 24 h.

### 2.8. Carrageenan-Induced Paw Edema

Tests were performed on male ICR mice, with six individuals in each group. A peptide sample was dissolved in sterile saline and administered intravenously at doses of 0.1 and 1.0 mg/kg. Control animals received an equivalent volume of sterile saline. Indomethacin at a dose of 5 mg/kg was used as a positive control and administered orally to animals. Each mouse received 20 μL of a 1% solution of carrageenan in the hind paw pad after 30 min in the case of saline and tested peptide and after 60 min in the case of indomethacin. Then, the resulting edema was measured at several time points (1, 3, 5, and 24 h) using a plethysmometer (Ugo Basile, Gemonio (VA), Italy).

### 2.9. Animals Euthanasia Procedure and Blood Sampling

Animals were terminally anaesthetized with sodium pentobarbital (40 mg per mouse *i.p*., Euthatal, Merial Animal Health, Essex, UK) 24 h after carrageenan injection. Then the thoracic cavity was opened and blood was collected in tubes with the ethylenediaminetetraacetic acid (EDTA) directly from the right atrium of the heart. The whole blood was centrifuged at 2.5 × 10^3^× *g* for 10 min to remove cells; the blood serum was then aliquoted and stored at −20 °C. These samples were analyzed for TNF-α in the enzyme-linked immunosorbent assay (ELISA) using a diagnostic kit according to the manufacturer’s protocol (CUSABIO BIOTECH Co., Ltd., Houston, TX, USA).

### 2.10. Molecular Modeling of Kunitz Peptides

The spatial structure models of HCRG1, HCRG2, HCRG21, and HCRG21 S5L were predicted with I-TASSER server [44] and analyzed using UCSF Chimera program (http://www.cgl.ucsf.edu/chimera) [45]. The ShPI-1 (PDB ID 1SHP) from the sea anemone *S. helianthus* was used as a template.

## 3. Results

### 3.1. Peptide Purification and Characterization

The peptides HCRG1 and HCRG2 were obtained following the final RP-HPLC step. According to MALDI-TOF/MS data, their molecular weights were 6196 and 6148 Da, respectively, which is consistent with our previously published results [38].

**Figure 1:**
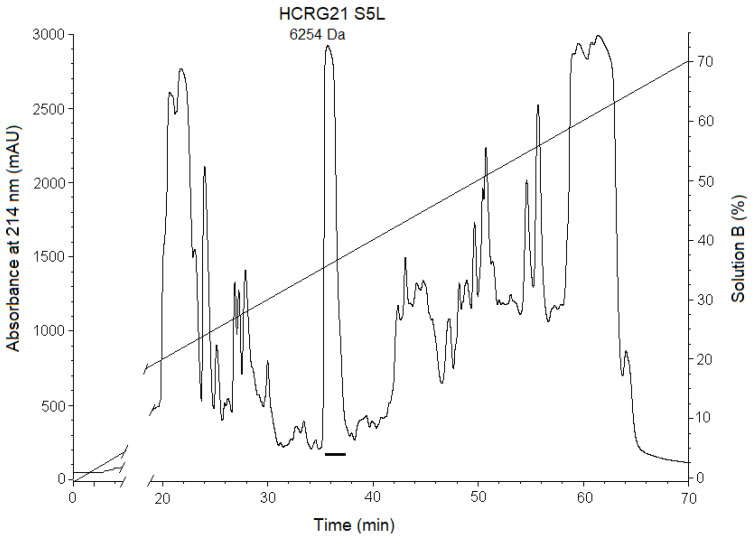
RP-HPLC elution profile of mutant peptide HCRG21 S5L, obtained as the result of hydrolysis of the fusion protein TRX-HCRG21 S5L by BrCN. The fraction containing the mature peptide is underlined. The measured molecular mass of HCRG21 S5L after RP-HPLC is indicated.

To obtain the mutant peptide HCRG21 S5L, a plasmid-based on pET32b-*hcrg*21 was generated using the site-directed mutagenesis technique. The target peptide was expressed and purified following the same conditions as reported for the recombinant peptide HCRG21 [23]. The retention time of HCRG21 S5L differed from that of HCRG21 and was 36 min (Figure 1). According to the MALDI-TOF/MS spectra, the molecular weight of the peptide was 6254 Da, which is consistent with the calculated value.

**Figure 2:**
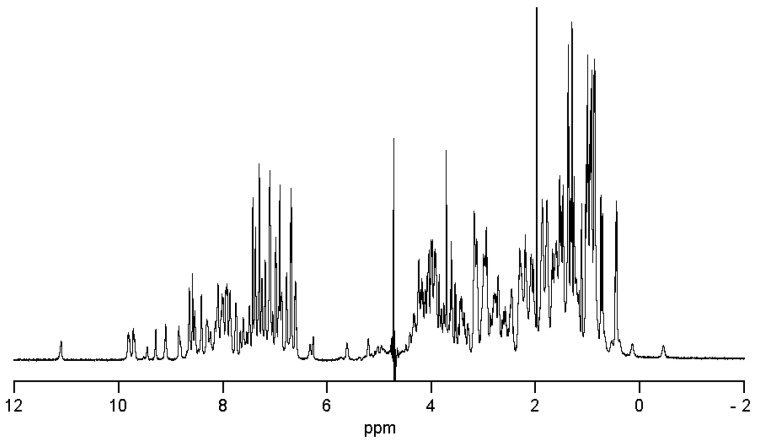
^1^H NMR spectrum of HCRG21 S5L.

To confirm the correct folding of the mutant peptide, the NMR spectroscopy technique was applied. The ^1^H NMR spectrum (Figure 2) indicates that peptide has a well-defined fold, as evidenced by the presence of resonance signals below 0 ppm and a wide chemical shift dispersion of amide hydrogens (9–6 ppm).

**Figure 3:**
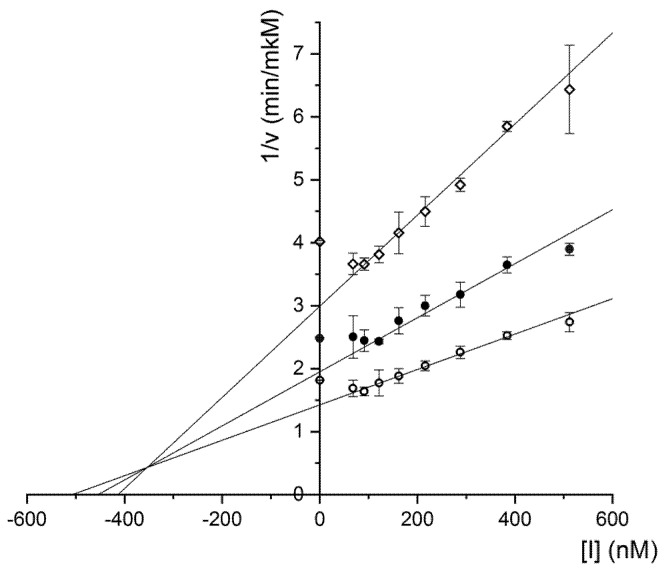
Determination of HCRG21 S5L Ki for trypsin by Dixon method. Substrate concentrations were 0.1 (◊), 0.16 (●), and 0.256 (○) mM. The constants were calculated based on the results of three independent experiments (n ≥ 3).

Peptide HCRG21 S5L was assayed for inhibitory activity against trypsin. The mutant peptide inhibited the trypsin activity with a *K*i value of 3.4 × 10^−7^ M (Figure 3) similar to HCRG21 (2 × 10^−7^ M [23]) and one order higher than for HCRG1 and HCRG2 (2.8 × 10^−8^ and 5 × 10^−8^ M, respectively [38]). This is due to the substitution of Thr to Lys in the P1 position which is functionally important for the serine protease inhibition [8,41,46].

**Figure 4:**
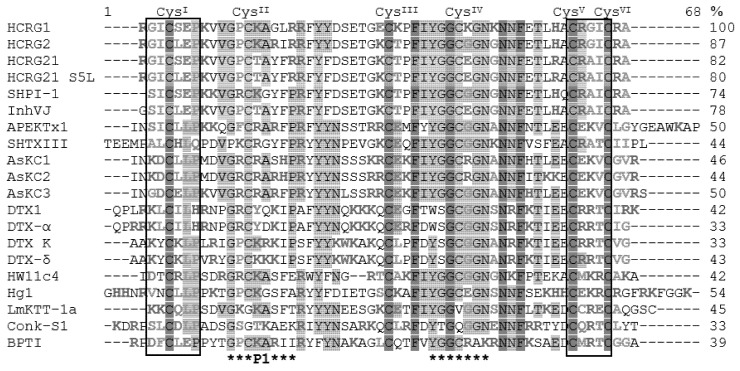
Multiple alignment of Kunitz peptides, blockers of Kv channels and BPTI. APEKTx1 (P61541) from the sea anemone *A. elegantissima*, AsKC1–AsKC3 (Q9TWG0, Q9TWF9, Q9TWF8) from *A. sulcata*, ShPI-1 (P31713) from *S. helianthus*, SHTXIII (B1B5I8) from *S. haddoni*, HCRG1, HCRG2, HCRG21, HCRG21 S5L, and InhVJ from *H. crispa*, HW11c4 (A0A023WBH6) from spider *Ornithoctonus huwena*; LmKTT-1a (P0DJ46) from scorpion *Lychas mucronatus*, Hg1 (P0C8W3) from *Hadrurus gertschi*; DTX1 (P00979), DTX-K (P00981) from snake *Dendroaspis polylepis*, DTX-α (P00980), DTX-δ (P00982) from *Dendroaspis angusticeps*; Conk-S1 (P0C1X2) from cone snail *Conus striatus*; BPTI (P00974) from bovine *Bos taurus*. Identical amino acids are shownondark-grey and conservative on light-gray background. Hydrophobic amino acids are indicated by green, positively charged by blue, negatively charged by red, and polar non-charged by pink letters. Frames highlight regions responsible for interaction with Kv channels [19]. Asterisks indicate a reactive site with P1 residue and site of weak interaction with serine proteases.

Comparison of the amino acid sequences of Kv toxins adopting the Kunitz-type fold showed that almost all these peptides contained six Cys residues in a typical pattern (I–VI, II–IV, III–V), except for the scorpion toxin LmKTT-1a and the cone snail toxin Conk-S1, which only had four residues (Figure 4). The sequence identities varied from 44% to 87% for sea anemone toxins and from 33% to 51% for snake, cone snail, spider, and scorpion toxins.

Garcia-Fernandez R. et al. suggested that functionally important amino acid residues for Kv blocking activity in the Kunitz-type toxin sequences were located in the N- and C-terminal parts of the molecule, in particular around CysI and CysV−CysVI, respectively (Figure 4) [19]. The differences in HCRG1 and HCRG2 were limited only by point substitutions: Ser5Leu, Gly16Arg, Glu28Lys, Lys30Thr, Lys38Gly, and Lys41Gly. Noteworthy, amino acid substitutions of HCRG1 near CysIII and CysIV, Glu28Lys, Lys30Thr, Lys38Gly, and Lys41Gly, were non-conservative replacement in comparison with HCRG2, which might influence the functional activity of these peptides. The main differences between HCRG1, HCRG2, HCRG21, and its mutant were three substitutions: Ser5Leu, Lys14Thr, and Lys/Gly38Glu.

### 3.2. Electrophysiological Experiments

**Figure 5:**
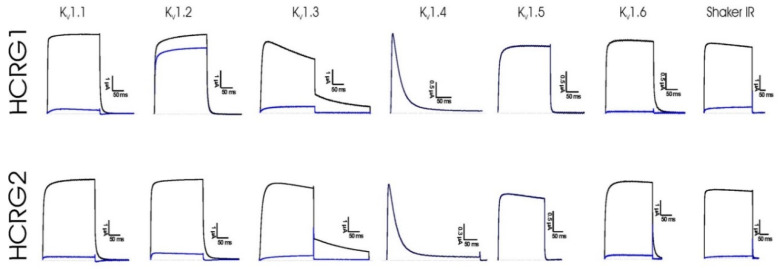
Electrophysiological analysis of HCRG1 and HCRG2 activities on several cloned voltage-gated potassium channel isoforms expressed in *X. laevis* oocytes. Representative whole-cell current traces in control and toxin conditions are shown. The blue line marks steady-state current traces after the application of 1 μM of peptides. Traces shown are representative traces of at least three independent experiments (n ≥ 3).

**Figure 6:**
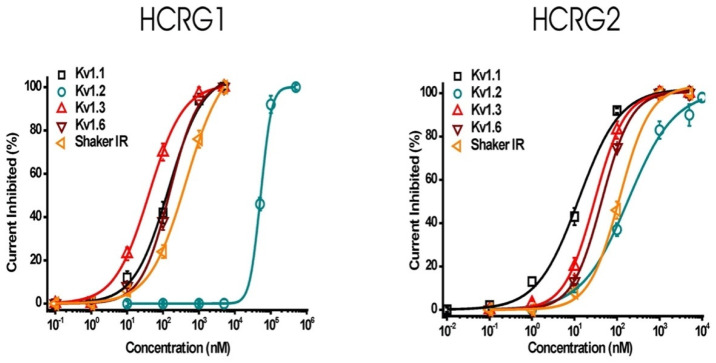
Dose–response curves of HCRG1 and HCRG2 on Kv1.1, Kv1.2, Kv1.3, Kv1.6, and *Shaker* IR channels obtained by plotting the percentage of blocked current as a function of increasing toxin concentrations. Traces shown are representative traces of at least three independent experiments (n ≥ 3).

The native peptides HCRG1 and HCRG2 were screened on a panel of six mammalian voltage-gated potassium (Kv1.1–Kv1.6) and the insect *Shaker* IR channels expressed in *X. laevis* oocytes. Electrophysiological testing revealed that the peptides, at a concentration 1 μM, inhibited approximately 100% of the Kv1.1, Kv1.3, Kv1.6, and *Shaker* IR channel currents. Interestingly, the potassium current through Kv1.2 channels was inhibited by HCRG1 and HCRG2 with 10% and 80% respectively (Figure 5). Notably, HCRG1 and HCRG2 appeared to be the first Kunitz-type peptides from sea anemones blocking Kv1.3. According to the dose–response curves, peptides HCRG1 and HCRG2 mainly differed in binding affinity to the Kv1.2 (Figure 6, Table 1).

**Figure 7:**
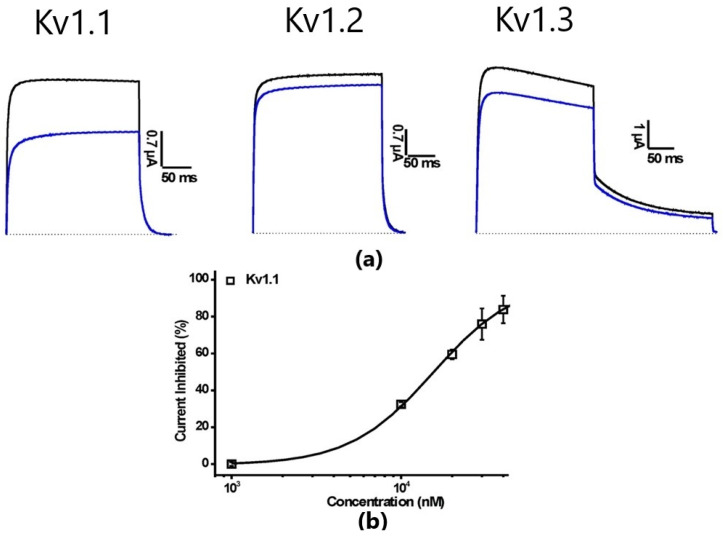
(**a**) Electrophysiological analysis of HCRG21 S5L activity on Kv1.1–Kv1.3 isoforms expressed in *X. laevis* oocytes. Representative whole-cell current traces in control and toxin conditions are shown. The blue line marks steady-state current traces after the application of 10 μM of peptide. (**b**) Dose–response curve for HCRG21 S5L on Kv1.1 channels. Traces shown are representative traces of at least three independent experiments (n ≥ 3).

We tested the mutant peptide HCRG21 S5L on Kv1.1, Kv1.2, and Kv1.3 channels. It was found that, in comparison with HCRG21 which did not exert any activity on the Kv channels, the mutant peptide at a concentration of 10 μM blocked Kv1.1, Kv1.2, and Kv1.3 currents with approximately 33%, 11%, and 14%, respectively (Figure 7). The IC_50_ value on Kv1.1 was much higher than the value obtained for HCRG1 and HCRG2 (Table 1).

### 3.3. Anti-Inflammatory Activity of HCRG1

Carrageenan-induced paw edema is widely used as an in vivo acute inflammatory response model [51]. Since HCRG1 is more specific to Kv1.3, it was tested in a model of acute local inflammation induced by carrageenan administration into mice paws. Before testing, we studied HCRG1 acute intravenous toxicity. After administration of the peptide, the animals exhibited normal behavior and external signs of intoxication (convulsions, asphyxia) or mortality were not detected. 

**Figure 8:**
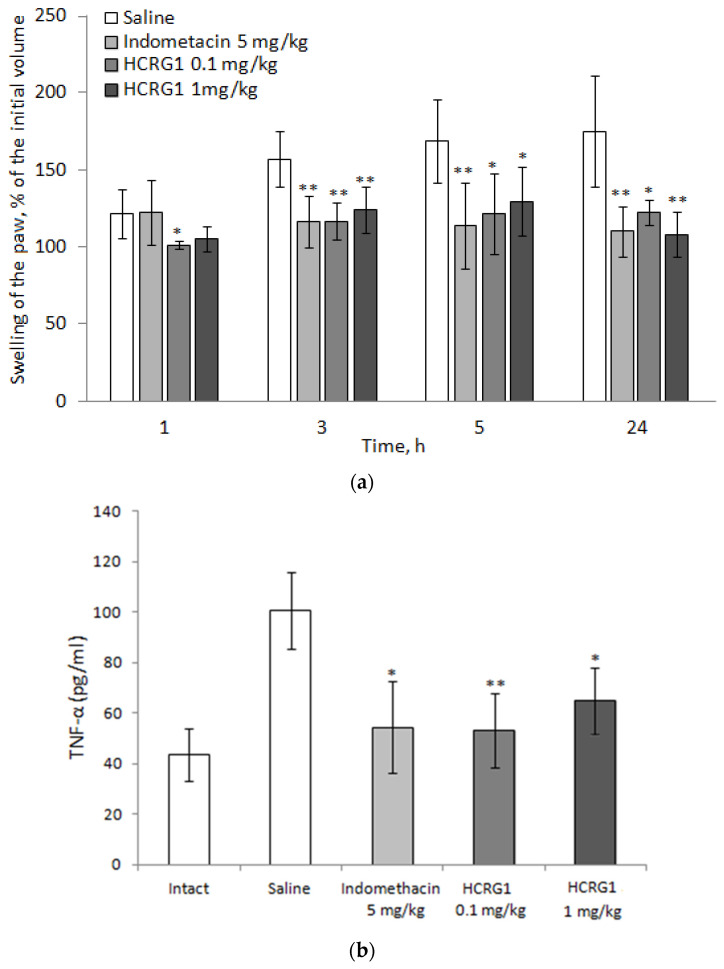
Effect of peptide HCRG1 on (**a**) paw swelling and (**b**) TNF-α production in mice with acute local inflammation induced by carrageenan administration. Control animals received a similar volume of saline (negative control) or indomethacin solution at a dose of 5 mg/kg (positive control). Intact animals on (**b**) were not subjected to any manipulation. The reliability of differences is calculated by the Student’s t-criterion. The value * *p* < 0.05, ** *p* < 0.01 is considered reliable in comparison with the saline group.

It was found that HCRG1 at doses of 0.1 and 1 mg/kg reduced the volume of developing edema during 24 h. Its effect was close to that of the nonsteroidal anti-inflammatory drug, indomethacin, at a dose of 5 mg/kg (Figure 8A). ELISA analysis of blood taken from animals showed that indomethacin and HCRG1 reduced the synthesis of TNF-α, a proinflammatory mediator that played a leading role in the development of edema and hyperalgesia in that model (Figure 8B) [51].

## 4. Discussion

Kunitz-type toxins are members of an ancient family that have been identified in many animal venoms, such as those of snakes, scorpions, spiders, cone snails, and sea anemones. Kunitz-type sea anemone type 2 toxins retained the ability to inhibit serine proteases which might be a venom defense mechanism against the prey’s proteases, similar to the toxins found in the venom of scorpions [52]. These peptides are believed to protect their own toxins from self-digestion by proteases. Moreover, they act synergistically with other peptide compounds of the venom and as such, they help to immobilize and kill the prey [1].

Up to now, more than a dozen Kunitz-type peptides produced by sea anemones of *Heteractis* genus (Stichodactylidae) have been described. Moreover, it has been determined that these peptides are encoded by a multigene superfamily composed of distinct GS-, GG-, GN-, and RG-gene families which are produced via a combinatorial library [8]. HCRG1, HCRG2, and HCRG21 are members of HCRG-family which includes 33 mature peptides. HCRG21 shares a high percentage of sequence identity with HCRG1 (82%) and HCRG2 (86%). Moreover, these peptides contain conserved amino acids at the N- and C-termini. Besides trypsin, Kunitz-type peptides from sea anemone *H. crispa* are also able to interact with other serine proteases (chymotrypsin, elastase, kallikrein) [19,39,53], modulate or block TRPV1 channel [22,23,24], revealing different kind of biological effects such as analgesic [22,54,55,56], anti-inflammatory [9,41,54,57], antihistamine [41,58], as well as neuroprotective activity [59,60]. However, despite their high degree of homology with the known bifunctional peptides, like kalicludines, SHTXIII, APEKTx1, and ShPI-1 [16,18,19], none of them has shown potassium channels blocking activity.

In this work, we identified a new activity of two previously characterized Kunitz peptides, HCRG1 and HCRG2, from *H. crispa* [38], using electrophysiological screening on six isoforms of Kv1 channels and insect *Shaker* IR channel expressed in *X. laevis* oocytes. Similar to toxin ShPI-1 from *S. helianthus* (Stichodactilidae) [19], HCRG1 and HCRG2 have also been shown to be active against more than one isoform of Kv1.x channels (Figure 5, (Table 1). The main difference compared to all sea anemone type 2 toxins is the ability of HCRG1 and HCRG2 to block Kv1.3 channels. Hence, and to the best of our knowledge, these peptides are the first Kunitz-type sea anemone toxins with activity towards Kv1.3 channels.

Among known Kunitz-type toxins produced by poisonous animals (Table 1), HCRG1 and HCRG2 turned out to be the least selective with respect to Kv1.x isoforms. Kunitz-type toxins from snake and scorpion venoms are more selective and usually modulate Kv1.x isoforms at lower concentrations (Table 1). Notably, HCRG1 inhibits Kv1.3 currents with an IC_50_ value of 40.7 nM, being about 3.5 and 1200 times more powerful blocker for it than for Kv1.1 and Kv1.2, respectively. As for HCRG2, the IC_50_ values differ by 2.5–6 times for all tested channels (Table 1). It is worth noticing that a large amount of a less selective peptide, identical to HCRG2, was found in the mucus of the closely related sea anemone *Heteractis magnifica* during proteomic analysis, which indicates its important place in the venom composition within the genus [9]. For many snake and scorpion toxins, as well as for sea anemone type 1 toxins with the Shk-fold, the amino acid determinants responsible for binding to Kv channels have already been identified. However, for sea anemone type 2 toxins, this question remains unresolved. The importance of a key basic residue (Lys or Arg) associated with a 6.6 ± 1 Å distant key hydrophobic residue (Leu, Tyr or Phe), together with a functional ring of basic amino acids, has been established [17]. Nevertheless, there are known examples of toxins lacking the dyad that still demonstrate blocking activities against Kv channels, suggesting that other epitopes are involved in the high-affinity interaction between the toxin and its target [17,61,62]. It seems that for type 2 toxins with the Kunitz fold, the number and distribution of charged, hydrophobic, and polar uncharged residues are important.

**Figure 9:**
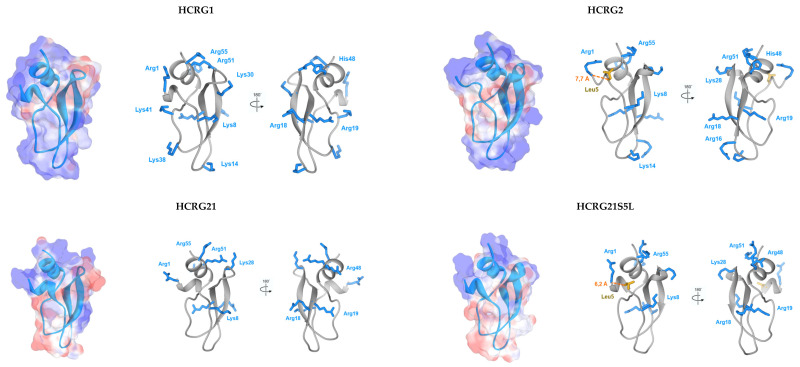
Spatial structures of HCRG1, HCRG2, HCRG21, and HCRG21S5L. 3D-Models of peptides are represented as a ribbon diagram with translucent surfaces accessible to the solvent, painted in accordance with the electrostatic potential: blue indicates the region of positive values, red—negative and gray—neutral. The side chains of positive (Arg and Lys) and hydrophobic (Leu5) amino acid residues are shown as sticks. The distance between the α-carbons of the above amino acid residues is indicated. The ShPI-1 (PDB ID 1SHP) from the sea anemone *S. helianthus* was used as a template. 3D models were made using Discovery Studio and UCSF Chimera.

Functionally important amino acid residues in the sequences of sea anemones and other venomous animals Kunitz-type toxins are located in the N- and C-terminal regions of the molecule, in particular around CysI and CysV−CysVI respectively [19]. These amino acid residues form a molecular recognition surface for interaction with Kvs, thanks to the conservative disulfide bond CysI–CysVI which brings together the N- and C-terminal regions of the molecule. Thus, Arg1, Ser5 or Leu5, Arg51, and Arg55 can be responsible for the activity of HCRG1 and HCRG2 (Figure 9). The side chain of Arg1, similar to Arg5 of the peptide Hg1 and Arg4 of the peptides DTX1 and DTX-α, can also make a significant contribution to the binding to Kv. However, HCRG21 and InhVJ which have the same amino acid residues at the indicated positions as HCRG1, HCRG2, and ShPI-1, do not demonstrate activity against Kv channels. Apparently, this is due to the presence in these peptide sequences of the residues Gly1 (for InhVJ), Thr14 and Glu38 (for both) (Figure 4) that impede interaction with the studied Kv channels and, possibly, make them specific to other ion channels [19].

In the HCRG1 and HCRG2 sequences, unlike those of the Kunitz type peptides AsKC1 and AsKC2 from *A. sulcata*, there is no distinct key residue identifiable for the interaction with Kv channel epitopes, similar to the dyad Lys5/Leu9 typical for dendrotoxins [13,47]. It can be surmised that this role might be partially fulfilled by Arg1/Leu5 residues in HCRG2 (Figure 9), since the affinity of this peptide to Kv1.1 is an order of magnitude higher than for HCRG1. HCRG21 is a full blocker of the TRPV1 channel but completely inactive against Kv1.x channels [23]. Interestingly, a single point mutation, Ser5 to Leu5, introduced for HCRG21 the properties of a weak blocker of Kv1.1, Kv1.2, and Kv1.3 channels (Figure 7). Using site-directed mutagenesis and chemical synthesis, it has been shown that the dyad Lys5 and Leu9 of DTX-α are crucial for channel blockage activity of this toxin [13,47]. We believe that for the peptides HCRG2 and HCRG21 S5L, the Arg1 and Leu5 residues can play the role of such a dyad. According to the results of molecular modeling, these residues are separated by 7.7 Å (HCRG2) and 6.2 Å (HCRG21 S5L) (Figure 9).

Most likely, the higher affinity of HCRG1 for Kv1.3 among the other Kv1 channels is caused by the residues Glu28, Lys30, Lys38, and Lys41, which sets this peptide apart from other sea anemone type 2 toxins (Figure 4). It has been well-established that there is a difference between residues forming the selective channel filter of Kv1.x channels: Asp377-Met378-Tyr379 for Kv1.1, Asp377-Met378-Val379 for Kv1.2, and Asp377-Met378-Hys379 for Kv1.3. The selectivity of different toxins to Kv1.x channel isoforms is dictated by the nature of the residue at position 379 [63]. Structural and functional studies using the site-directed mutagenesis method will further determine the functional significance of the designated amino acids of HCRG1 and HCRG2.

Kv1.3 channels have gained a prominent role for their possibility to control neuroinflammatory and autoimmune diseases [33,64,65,66]. Inflammation involves several processes including the activation of inflammatory cells, the secretion of pro-inflammatory cytokines and the release of various inflammatory mediators, leading to symptoms of inflammation such as redness, swelling, fever and pain [67]. Previously, we have shown that the peptides HCRG1 and HCRG2 are able to reduce the synthesis of pro-inflammatory mediators, pro-IL-1β, IL-6, and TNF-α, induced by the addition of bacterial lipopolysaccharide to J774A.1 macrophages [36]. These effects can be achieved by inhibiting the proteases linked to inflammatory processes, as well as by blocking of Kv1.3 channels. On one hand, an anti-inflammatory effect was shown for BPTI, known as an inhibitor of different serine proteases, and bikunin, a human Kunitz-type peptide that inhibits the production of thromboxane B2, TNF-α, and IL-8 in macrophages treated with LPS [68]. On the other hand, it has been established that the treatment of autoreactive T-lymphocytes by ShK-186 (analog of ShK from *S. helianthus*) decreased the levels of IL-2, IL-4, interferon-γ, and TNF-α [37]. In this work, we have shown that HCRG1 mice pretreatment (at doses of 0.1 and 1 mg/kg) significantly reduces (~40%) paw edema during 24 h after carrageenan administration. In addition, HCRG1 at a dose of 0.1 mg/kg inhibits the synthesis of TNF-α similar to indomethacin after 24 h (Figure 8B). These data indicate that HCRG1 has an anti-inflammatory effect by inhibiting the secretion of TNF-α, a pro-inflammatory mediator that demonstrates a leading role in the development of edema and hyperalgesia in this model.

In summary, we found out that HCRG1 and HCRG2 from the sea anemone *H. crispa* are new representatives of type 2 toxins demonstrating Kv inhibitory activity similar to other members. Furthermore, they are the first Kunitz-type peptides blocking the activity of prospect pharmacological channel Kv1.3. We first showed the ability of Kunitz-type peptides with dual inhibitory activity, namely towards Kv and serine proteases, to demonstrate anti-inflammatory effects during acute inflammation. We cannot clearly conclude which of the two activities results in the observed effect, but presumably, both can contribute or enhance the peptide action in the organism. Artificial mutant HCRG21 S5L is a curious example of how the substitution of one amino acid residue changes the specificity of sea anemone Kunitz-type toxin from the channel of TRP to Kv family. It shows a fine line between a specific inhibitor of TRPV1 channel and a toxin with a broader function. This manuscript is the starting point for a deeper investigation of the importance of single amino acid residues and the establishment of the evolutionary patterns of Kunitz-type peptides from sea anemones, so similar in their amino acid sequences and so different in the activities.

## Figures and Tables

**Table 1:** Kunitz-type voltage-gated potassium channel toxins from animal venoms.

Peptide	IC_50_ (nM)					Reference
	Kv1.1	Kv1.2	Kv1.3	Kv1.6	*Shaker* IR	
**Sea anemones**						
HCRG1	142.6 ± 28.1	52,199.0 ± 2751.7	40.7 ± 4.1	154.9 ± 20.4	433.1 ± 43.9	This work
HCRG2	12.6 ± 1.72	181.7 ± 38.5	29.7 ± 1.3	43.9 ± 1.3	114.9 ± 13.9	
HCRG21	-	-	-	-	-	[23]
HCRG21 S5L	15,600 ± 0.24	-	-	n.d.	n.d.	This work
ShPI-1	117 ± 15	9 ± 2	-	9 ± 2	-	[19]
APEKTx1	0.9	-	-	-	-	[17]
AsKC1	n.d.	2800	n.d.	n.d.	n.d.	[16]
AsKC2	n.d.	1100	n.d.	n.d.	n.d.	[16]
AsKC3	n.d.	1300	n.d.	n.d.	n.d.	[16]
SHTXIII	270 *	270 *	n.d.	270 *	n.d.	[18]
**Snakes**						
DTX-α	1.1	0.4	-	9	n.d.	[47]
DTX-I	3.1	0.13	n.d.	+	n.d.	[48]
DTX K	0.03	-	n.d.	-	n.d.	[48]
DTX-δ	0.01	n.d.	n.d.	n.d.	1000	[49]
**Cone snail**						
Conk-S1	n.d.	n.d.	n.d.	n.d.	1.33	[15]
**Scorpions**						
LmKTT-1a	>1000	>1000	1580 ± 73	n.d.	n.d.	[50]
Hg1	-	-	6.2	-	-	[10]
**Spider**						
HW11c4	>10, 000	-	-	n.d.	n.d.	[14]

* ^125^I α-DTX dendrotoxin binding to synaptosomal membranes, n.d. not determined, - no or weak activity.

## References

[1] Mourão C.B.F., Schwartz E.F. (2013). Protease inhibitors from marine venomous animals and their counterparts in terrestrial venomous animals. Mar. Drugs.

[2] Kunitz M., Northrop J.H. (1936). Isolation from beef pancreas of crystalline trypsinogen, trypsin, a trypsin inhibitor, and an inhibitor-trypsin compound. J. Gen. Physiol..

[3] Ascenzi P., Bocedi A., Bolognesi M., Spallarossa A., Coletta M., De Cristofaro R., Menegatti E. (2003). The bovine basic pancreatic trypsin inhibitor (Kunitz inhibitor): A milestone protein. Curr. Protein Pept. Sci..

[4] Sun Z., Lu W., Jiang A., Chen J., Tang F., Liu J.-N. (2009). Expression, purification and characterization of aprotinin and a human analogue of aprotinin. Protein Expr. Purif..

[5] Buczek O., Koscielska-Kasprzak K., Krowarsch D., Dadlez M., Otlewski J. (2002). Analysis of serine proteinase-inhibitor interaction by alanine shaving. Protein Sci..

[6] Dai S.-X., Zhang A.-D., Huang J.-F. (2012). Evolution, expansion and expression of the Kunitz/BPTI gene family associated with long-term blood feeding in Ixodes Scapularis. BMC Evol. Biol..

[7] Yuan C.H., He Q.Y., Peng K., Diao J.B., Jiang L.P., Tang X., Liang S.P. (2008). Discovery of a distinct superfamily of kunitz-type toxin (KTT) from Tarantulas. PLoS ONE.

[8] Isaeva M.P., Chausova V.E., Zelepuga E.A., Guzev K.V., Tabakmakher V.M., Monastyrnaya M.M., Kozlovskaya E.P. (2012). A new multigene superfamily of Kunitz-type protease inhibitors from sea anemone *Heteractis crispa*. Peptides.

[9] Sintsova O., Gladkikh I., Chausova V., Monastyrnaya M., Anastyuk S., Chernikov O., Yurchenko E., Aminin D., Isaeva M., Leychenko E. (2018). Peptide fingerprinting of the sea anemone *Heteractis magnifica* mucus revealed neurotoxins, Kunitz-type proteinase inhibitors and a new β-defensin α-amylase inhibitor. J. Proteomics.

[10] Chen Z.Y., Hu Y.T., Yang W.S., He Y.W., Feng J., Wang B., Zhao R.M., Ding J.P., Cao Z.J., Li W.X. (2012). Hg1, novel peptide inhibitor specific for Kv1.3 channels from first scorpion Kunitz-type potassium channel toxin family. J. Biol. Chem..

[11] Harvey A.L., Anderson A.J. (1985). Dendrotoxins: Snake toxins that block potassium channels and facilitate neurotransmitter release. Pharmacol. Ther..

[12] Lancelin J.M., Foray M.F., Poncin M., Hollecker M., Marion D. (1994). Proteinase inhibitor homologues as potassium channel blockers. Nat. Struct. Biol..

[13] Owen D.G., Hall A., Stephens G., Stow J., Robertson B. (1997). The relative potencies of dendrotoxins as blockers of the cloned expressed in Chinese hamster ovary cells. Br. J. Pharmacol..

[14] Jiang L., Deng M., Duan Z., Tang X., Liang S. (2014). Molecular cloning, bioinformatics analysis and functional characterization of HWTX-XI toxin superfamily from the spider *Ornithoctonus huwena*. Peptides.

[15] Bayrhuber M., Vijayan V., Ferber M., Graf R., Korukottu J., Imperial J., Garrett J.E., Olivera B.M., Terlau H., Zweckstetter M. (2005). Conkunitzin-S1 is the first member of a new Kunitz-type neurotoxin family: Structural and functional characterization. J. Biol. Chem..

[16] Schweitz H., Bruhn T., Guillemare E., Moinier D., Lancelin J.-M.M., Béress L., Lazdunski M., Beress L., Lazdunski M., Béress L. (1995). Kalicludines and Kaliseptine: Two different classes of sea anemone toxins for voltage sensitive K+ cannels. J. Biol. Chem..

[17] Peigneur S., Billen B., Derua R., Waelkens E., Debaveye S., Béress L., Tytgat J. (2011). A bifunctional sea anemone peptide with Kunitz type protease and potassium channel inhibiting properties. Biochem. Pharmacol..

[18] Honma T., Kawahata S., Ishida M., Nagai H., Nagashima Y., Shiomi K. (2008). Novel peptide toxins from the sea anemone *Stichodactyla haddoni*. Peptides.

[19] García-Fernández R., Peigneur S., Pons T., Alvarez C., González L., Chávez M.A., Tytgat J. (2016). The kunitz-type protein ShPI-1 inhibits serine proteases and voltage-gated potassium channels. Toxins (Basel).

[20] Schweitz H., Heurteaux C., Bois P., Moinier D., Romey G., Lazdunski M. (1994). Calcicludine, a venom peptide of the Kunitz-type protease inhibitor family, is a potent blocker of high-threshold Ca2+ channels with a high affinity for L-type channels in cerebellar granule neurons. Proc. Natl. Acad. Sci. USA.

[21] Báez A., Salceda E., Fló M., Graña M., Fernández C., Vega R., Soto E. (2015). α-Dendrotoxin inhibits the ASIC current in dorsal root ganglion neurons from rat. Neurosci. Lett..

[22] Andreev Y.A., Kozlov S.A., Koshelev S.G., Ivanova E.A., Monastyrnaya M.M., Kozlovskaya E.P., Grishin E.V. (2008). Analgesic compound from sea anemone *Heteractis crispa* is the first polypeptide inhibitor of vanilloid receptor 1 (TRPV1). J. Biol. Chem..

[23] Monastyrnaya M., Peigneur S., Zelepuga E., Sintsova O., Gladkikh I., Leychenko E., Isaeva M., Tytgat J., Kozlovskaya E. (2016). Kunitz-Type Peptide HCRG21 from the Sea Anemone *Heteractis crispa* Is a Full Antagonist of the TRPV1 Receptor. Mar. Drugs.

[24] Nikolaev M.V., Dorofeeva N.A., Komarova M.S., Korolkova Y.V., Andreev Y.A., Mosharova I.V., Grishin E.V., Tikhonov D.B., Kozlov S.A. (2017). TRPV1 activation power can switch an action mode for its polypeptide ligands. PLoS ONE.

[25] Papers J.B.C., Doi M., Mans B.J., Louw A.I., Neitz A.W.H. (2002). Savignygrin, a Platelet Aggregation Inhibitor from the Soft Tick *Ornithodoros savignyi*, Presents the RGD Integrin Recognition Motif on the Kunitz-BPTI Fold. J. Biol. Chem..

[26] Ciolek J., Reinfrank H., Sigismeau S., Mouillac B., Peigneur S., Tytgat J., Droctov L., Mourier G., De Pauw E., Servent D. (2017). Green mamba peptide targets type-2 vasopressin receptor against polycystic kidney disease. Proc. Natl. Acad. Sci. USA.

[27] Orts D.J.B., Moran Y., Cologna C.T., Peigneur S., Madio B., Praher D., Quinton L., De Pauw E., Bicudo J.E.P.W., Tytgat J. (2013). BcsTx3 is a founder of a novel sea anemone toxin family of potassium channel blocker. FEBS J..

[28] Madio B., Peigneur S., Chin Y.K.Y., Hamilton B.R., Henriques S.T., Smith J.J., Cristofori B., Zoltan A., Berin D., Paul A.B. (2018). PHAB toxins: A unique family of predatory sea anemone toxins evolving via intra—Gene concerted evolution defines a new peptide fold. Cell. Mol. Life Sci..

[29] Gutman G.A., Chandy K.G., Grissmer S., Lazdunski M., Mckinnon D., Pardo L.A., Robertson G.A., Rudy B., Sanguinetti M.C., Stu W. (2005). International Union of Pharmacology. LIII. Nomenclature and Molecular Relationships of Voltage-Gated Potassium Channels. Pharmacol. Rev..

[30] Norton R.S., Chandy K.G. (2017). Venom-derived peptide inhibitors of voltage-gated potassium channels. Neuropharmacology.

[31] Finol-Urdaneta R.K., Belovanovic A., Micic-Vicovac M., Kinsella G.K., McArthur J.R., Al-Sabi A. (2020). Marine toxins targeting Kv1 channels: Pharmacological tools and therapeutic scaffolds. Mar. Drugs.

[32] Pérez-Verdaguer M., Capera J., Serrano-Novillo C., Estadella I., Sastre D., Felipe A. (2016). The voltage-gated potassium channel Kv1.3 is a promising multitherapeutic target against human pathologies. Expert Opin. Ther. Targets.

[33] Rangaraju S., Chi V., Pennington M.W., Chandy K.G. (2009). Kv1.3 Potassium Channels as a Therapeutic Target in Multiple Sclerosis. Expert Opin. Ther. Targets.

[34] Tajti G., Wai D.C.C., Panyi G., Norton R.S. (2020). The voltage-gated potassium channel KV1.3 as a therapeutic target for venom-derived peptides. Biochem. Pharmacol..

[35] Castañeda O., Sotolongo V., Amor A.M., Stöcklin R., Anderson A.J., Harvey A.L., Engström A., Wernstedt C., Karlsson E. (1995). Characterization of a potassium channel toxin from the Caribbean Sea anemone *Stichodactyla helianthus*. Toxicon.

[36] Shen B., Cao Z., Li W., Sabatier J.-M.M., Wu Y. (2017). Treating autoimmune disorders with venom-derived peptides. Expert Opin. Biol. Ther..

[37] Chi V., Pennington M.W., Norton R.S., Tarcha E.J., Londono L.M., Sims-Fahey B., Upadhyay S.K., Lakey J.T., Iadonato S., Wulff H. (2012). Development of a sea anemone toxin as an immunomodulator for therapy of autoimmune diseases. Toxicon.

[38] Gladkikh I., Monastyrnaya M., Zelepuga E., Sintsova O., Tabakmakher V., Gnedenko O., Ivanov A., Hua K.-F., Kozlovskaya E. (2015). New kunitz-type HCRG polypeptides from the sea anemone *Heteractis crispa*. Mar. Drugs.

[39] Gladkikh I., Monastyrnaya M., Leychenko E., Zelepuga E., Chausova V., Isaeva M., Anastyuk S., Andreev Y., Peigneur S., Tytgat J. (2012). Atypical reactive center Kunitz-type inhibitor from the sea anemone *Heteractis crispa*. Mar. Drugs.

[40] Hwang T.L., Shaka A.J. (1995). Water Suppression That Works. Excitation Sculpting Using Arbitrary Wave-Forms and Pulsed-Field Gradients. J. Magn. Reson. Ser. A.

[41] Sintsova O.V., Monastyrnaya M.M., Pislyagin E.A., Menchinskaya E.S., Leychenko E.V., Aminin D.L., Kozlovskaya E.P. (2015). Anti-inflammatory activity of a polypeptide from the *Heteractis crispa* sea anemone. Russ. J. Bioorg. Chem..

[42] Dixon M. (1953). The determination of enzyme inhibitor constants. Biochem. J..

[43] Liman E.R., Tytgat J., Hess P. (1992). Subunit stoichiometry of a mammalian K+ channel determined by construction of multimeric cDNAs. Neuron.

[44] Yang J., Zhang Y. (2015). I-TASSER server: New development for protein structure and function predictions. Nucleic Acids Res..

[45] Pettersen E.F., Goddard T.D., Huang C.C., Couch G.S., Greenblatt D.M., Meng E.C., Ferrin T.E. (2004). UCSF Chimera—A visualization system for exploratory research and analysis. J. Comput. Chem..

[46] Helland R., Otlewski J., Sundheim O., Dadlez M., Smalås A.O. (1999). The crystal structures of the complexes between bovine beta-trypsin and ten P1 variants of BPTI. J. Mol. Biol..

[47] Harvey A.L. (2001). Twenty years of dendrotoxins. Toxicon.

[48] Robertson B., Owen D., Stow J., Butler C., Newland C. (1996). Novel effects of dendrotoxin homologues on subtypes of mammalian Kv1 potassium channels expressed in Xenopus oocytes. FEBS Lett..

[49] Imredy J.P., MacKinnon R. (2000). Energetic and structural interactions between δ-dendrotoxin and a voltage-gated potassium channel. J. Mol. Biol..

[50] Chen Z., Luo F., Feng J., Yang W., Zeng D., Zhao R., Cao Z., Liu M., Li W., Jiang L. (2013). Genomic and Structural Characterization of Kunitz-Type Peptide LmKTT-1a Highlights Diversity and Evolution of Scorpion Potassium Channel Toxins. PLoS ONE.

[51] Rocha A.C.C., Fernandes E.S., Quintão N.L.M., Campos M.M., Calixto J.B. (2006). Relevance of tumour necrosis factor-α for the inflammatory and nociceptive responses evoked by carrageenan in the mouse paw. Br. J. Pharmacol..

[52] Ma H., Xiao-Peng T., Yang S.-L., Lu Q.-M., Lai R. (2016). Protease inhibitor in scorpion (*Mesobuthus eupeus*) venom prolongs the biological activities of the crude venom. Chin. J. Nat. Med..

[53] Kvetkina A.N., Kaluzhskiy L.A., Leychenko E.V., Isaeva M.P. (2019). New Targets of Kunitz-Type Peptide from Sea Anemone *Heteractis magnifica*. Dokl. Biochem. Biophys..

[54] Andreev Y.A., Kozlov S.A., Korolkova Y.V., Dyachenko I.A., Bondarenko D.A., Skobtsov D.I., Murashev A.N., Kotova P.D., Rogachevskaja O.A., Kabanova N.V. (2013). Polypeptide modulators of TRPV1 produce analgesia without hyperthermia. Mar. Drugs.

[55] Tabakmakher V.M., Sintsova O.V., Krivoshapko O.N., Zelepuga E.A., Monastyrnaya M.M., Kozlovskaya E.P. (2015). Analgesic effect of novel Kunitz-type polypeptides of the sea anemone *Heteractis crispa*. Dokl. Biochem. Biophys..

[56] Sintsova O.V., Palikov V.A., Palikova Y.A., Klimovich A.A., Gladkikh I.N., Andreev Y.A., Monastyrnaya M.M., Kozlovskaya E.P., Dyachenko I.A., Kozlov S.A. (2020). Peptide Blocker of Ion Channel TRPV1 Exhibits a Long Analgesic Effect in the Heat Stimulation Model. Dokl. Biochem. Biophys..

[57] Sokotun I.N., Gnedenko O.V., Leychenko A.V., Monastyrnaya M.M., Kozlovskaya E.P., Molnar A.A., Ivanov A.S., Gnedenko O.V., Leychenko E.V., Monastyrnaya M.M. (2007). Study of the interaction of trypsin inhibitor from the sea anemone *Radianthus macrodactylus* with proteases. Biochem. Suppl. Ser. B Biomed. Chem..

[58] Sintsova O.V., Pislyagin E.A., Gladkikh I.N., Monastyrnaya M.M., Menchinskaya E.S. (2017). Kunitz-Type Peptides of the Sea Anemone Heteractis crispa: Potential Anti-Inflammatory Compounds. Russ. J. Bioorg. Chem..

[59] Kvetkina A.N., Leychenko E.V., Yurchenko E.A., Pislyagin E.A., Peigneur S., Tytgat J., Isaeva M., Aminin D., Kozlovskaya E.P. (2018). A New Iq-Peptide of the Kunitz Type from the *Heteractis magnifica* Sea Anemone Exhibits Neuroprotective Activity in a Model of Alzheimer’s Disease. Russ. J. Bioorg. Chem..

[60] Kvetkina A., Leychenko E., Chausova V., Zelepuga E., Chernysheva N., Guzev K., Pislyagin E., Yurchenko E., Menchinskaya E., Aminin D. (2020). A new multigene HCIQ subfamily from the sea anemone *Heteractis crispa* encodes Kunitz-peptides exhibiting neuroprotective activity against 6-hydroxydopamine. Sci. Rep..

[61] Shon K.J., Stocker M., Terlau H., Stühmer W., Jacobsen R., Walker C., Grilley M., Watkins M., Hillyard D.R., Gray W.R. (1998). κ-Conotoxin PVIIA is a peptide inhibiting the Shaker K+ channel. J. Biol. Chem..

[62] Huys I., Xu C.Q., Wang C.Z., Vacher H., Martin-Eauclaire M.F., Chi C.W., Tytgat J. (2004). BmTx3, a scorpion toxin with two putative functional faces separately active on A-type K+ and HERG currents. Biochem. J..

[63] Gilquin B., Braud S., Eriksson M.A.L., Roux B., Bailey T.D., Priest B.T., Garcia M.L., Ménez A., Gasparini S. (2005). A variable residue in the pore of Kv1 channels is critical for the high affinity of blockers from sea anemones and scorpions. J. Biol. Chem..

[64] Wang X., Li G., Guo J., Zhang Z., Zhang S., Zhu Y., Cheng J., Yu L., Ji Y., Tao J. (2020). Kv1.3 Channel as a Key Therapeutic Target for Neuroinflammatory Diseases: State of the Art and Beyond. Front. Neurosci..

[65] Sarkar S., Nguyen H.M., Malovic E., Luo J., Langley M., Palanisamy B.N., Singh N., Manne S., Neal M., Gabrielle M. (2020). Kv1.3 modulates neuroinflammation and neurodegeneration in Parkinson’s disease. J. Clin. Investig..

[66] Rangaraju S., Raza S.A., Pennati A., Deng Q., Dammer E.B., Duong D., Pennington M.W., Tansey M.G., Lah J.J., Betarbet R. (2017). A systems pharmacology-based approach to identify novel Kv1.3 channel-dependent mechanisms in microglial activation. J. Neuroinflamm..

[67] Mansouri M.T., Hemmati A.A., Naghizadeh B., Mard S.A., Rezaie A., Ghorbanzadeh B. (2015). A study of the mechanisms underlying the anti-inflammatory effect of ellagic acid in carrageenan-induced paw edema in rats. Indian J. Pharmacol..

[68] Shigetomi H., Onogi A., Kajiwara H., Yoshida S., Furukawa N., Haruta S., Tanase Y., Kanayama S., Noguchi T., Yamada Y. (2010). Anti-inflammatory actions of serine protease inhibitors containing the Kunitz domain. Inflamm. Res..

